# 5-Fluorouracil Loaded Chitosan–PVA/Na^+^MMT Nanocomposite Films for Drug Release and Antimicrobial Activity

**DOI:** 10.1007/s40820-016-0086-4

**Published:** 2016-03-16

**Authors:** A. Babul Reddy, B. Manjula, T. Jayaramudu, E. R. Sadiku, P. Anand Babu, S. Periyar Selvam

**Affiliations:** 1 Department of Chemical, Metallurgical and Materials Engineering, Tshwane University of Technology, CSIR Campus, Building 14D, Lynwood Ridge, Private Bag X025, Pretoria, 0040 South Africa; 2 Department of Food Process Engineering, School of Bioengineering, SRM University, Kattankulathur, Tamil Nadu 603203 India

**Keywords:** Biopolymer, Chitosan–PVA/Na^+^MMT, Montmorillonite, 5-Fluorouracil, Drug release, Antimicrobial activity

## Abstract

In the present study, chitosan and polyvinyl alcohol (PVA) were blended with different concentrations of sodium montmorillonite (Na^+^MMT) clay solution by a solvent casting method. X-ray diffraction and transition electron microscope results show that the film properties are related to the co-existence of Na^+^MMT intercalation/exfoliation in the blend and the interaction between chitosan–PVA and Na^+^MMT. 5-Fluorouracil (5-FU) was loaded with chitosan–PVA/Na^+^MMT nanocomposite films for in vitro drug delivery study. The antimicrobial activity of the chitosan–PVA/Na^+^MMT films showed significant effect against *Salmonella* (*Gram*-*negative*) and *Staphylococcus aureus* (*Gram*-*positive*), whereas 5-FU encapsulated chitosan–PVA/Na^+^MMT bio-nanocomposite films did not show any inhibition against bacteria. Our results indicate that combination of a flexible and soft polymeric material with high drug loading ability of a hard inorganic porous material can produce improved control over degradation and drug release. It will be an economically viable method for preparation of advanced drug delivery vehicles and biodegradable implants or scaffolds.

## Introduction

In the past few decades, drug delivery systems have been of great interest and resulted in many efforts to realize the effectiveness and targeted drug delivery tendency as well as to reduce the associated side effects. Controlled drug delivery system is necessary in order to develop new nano-medicines. Thus, the carriers used for drug release are generally biodegradable polymers [1] and hard inorganic porous matrices [2]. In recent past, biodegradable polymer attracted much attention owing to its potential applications as a carrier in drug delivery systems.

Chitosan is a semi-crystalline and linear polysaccharide composed of (1–4)-2-acetamido-2-deoxy-*b*-d-glucan (*N*-acetyl d-glucosamine) and (1-4)-2-amino-2-deoxy-*b*-d-glucan (d-glucosamine) units. It is not widely present in the environment but can be easily obtained from the partial deacetylation of a natural polymer chitin [3]. The deacetylation degree of chitosan provides valuable information regarding to the number of amino groups (–NH_2_) along the chains and it can be measured as the ratio of d-glucosamine to the sum of d-glucosamine and *N*-acetyl d-glucosamine. For a chitosan, the deacetylated chitin must have at least 60 % of d-glucosamine residues [4] and controlled by chemical hydrolysis under harsh alkaline conditions or enzymatic hydrolysis in the presence of particular enzymes among of chitin deacetylase [5]. The presence of amino groups in the chitosan structure differentiates the chitosan from chitin and allows this polymer to have several unique properties. Undoubtedly, the amino groups of the d-glucosamine residues might be protonated, when it is soluble in aqueous acidic solutions (pH < 6). Whereas the applications of chitin are tremendously limited due to its weak solubility in water or other organic solvents. Interestingly, the aqueous acidic solubility of chitosan is pH dependent, permitting its processability under warm conditions, which opens the door for many applications, particularly in the field of pharmaceutical and cosmetics [3]. This polysaccharide has been extensively studied in the field of biomaterials because of its biodegradability, biocompatibility, and biological properties. Among various polymer development processes, polymer blending is one of the most economical and rapid ways to innovate novel materials with vital properties and it has made great scientific and commercial progresses [6].

Polyvinyl alcohol (PVA)-based nanocomposites are one of the familiar polymer composites that have been used in various biomedical applications (implants, artificial organs, contact lenses, drug delivery devices, wound dressings, etc.) due to its good biocompatibility behavior [7–11]. There are numerous methods available for crosslinking PVA chains to synthesize PVA composites, including bulk mixing with crosslinking agents such as glutaraldehyde (GA), freezing–thawing cyclic process [12], as well as electron beam irradiation [13].

Nowadays increased attention has been focused on drug intercalated smectites, particularly montmorillonite (MMT) pharmaceutical grade mineral clay [14, 15]. MMT has cation exchange capacity, good adsorption capacity, large specific surface area, and drug-carrying ability. It is hydrophilic and highly dispersible in water and can aid in the synthesis of a wide variety of hydrophilic and protonated organic molecules, which can be released in controlled fashion by replacement with other types of cations in the drug release processes [16–18]. Therefore, MMT is a good delivery carrier of hydrophilic drugs due to its high aspect ratio and can afford mucoadhesive ability for the nanoparticles to cross the gastrointestinal barrier [19, 20]. So far, MMT has been used as a controlled release system and proved to be nontoxic by hematological, biochemical and histopathological analyses in rat models [21]. It is also utilized as a sustained release carrier for various therapeutic molecules, such as 5-fluorouracil (5-FU) [22], sertraline [23], vitamin B1 [14, 15], promethazine chloride [24], and buspirone hydrochloride [25].

5-FU is an effective chemotherapy option available for the treatment of colorectal cancer [26, 27], stomach cancer [27], breast cancer [28], brain tumor [29, 30], liver cancer [31], pancreatic cancers [32–34], and lung cancer [35–38]. It is a pyrimidine analog that restrains the biosynthesis of deoxyribonucleotides for DNA replication through constraining thymidylate synthase activity, resulting to thymidine exhaustion, incorporation of deoxyuridine triphosphate into DNA and subsequently causing cell death [39–41]. However, 5-FU has limitations, such as short biological half-life due to rapid metabolism, non-uniform and incomplete oral absorption owing to metabolism by dihydropyrimidine dehydrogenase [42–45], toxic side effects on bone marrow and gastrointestinal tract, and non-selective action against healthy cells [46]. For successful cancer treatment, overcoming the toxic side effects on bone marrow is highly essential, which might possibly be achieved by the control release of the drug by intercalated in the clay interlayer and biopolymeric systems.

**Fig. 1:**
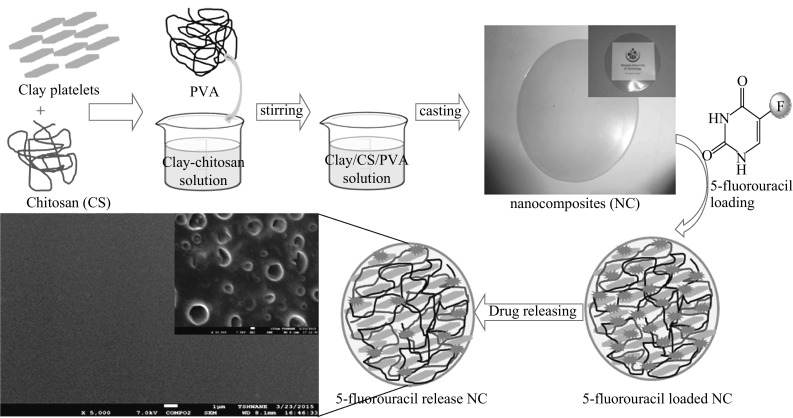
Schematic representation for the formation of 5-FU loaded chitosan/PVA–Na^+^MMT nanocomposites

However, no report is available in the literatures for combination of biodegradable polymer chitosan–PVA and Na^+^MMT for controlled release of 5-FU. In this study, we have tried to develop a biodegradable and biocompatible polymer to control drug release properties with pharmaceutical grade MMT in order to produce oral and controlled drug delivery formulations for 5-FU. Being a highly hydrophilic drug molecule, it is very difficult to encapsulate high amount of 5-FU within the hydrophilic polymer matrix. Therefore, in the present study, the modified solvent casting method has been developed to entrap substantial amount of drug in the synthesized formulations. The schematic representation of the process is shown in Fig. 1.

## Experimental

### Materials

High molecular weight chitosan (viscosity of between 800 and 2000 cps), PVA (96 % hydrolysed, molecular weight 85,000–145,000, and the degree of deacetylation higher than 75 %), and 5-FU (99 %) were sourced from Sigma-Aldrich (South Africa). GA, sodium chloride (NaCl), sodium hydroxide (NaOH), silver nitrate (AgNO_3_), acetic acid (CH_3_COOH), and de-ionized water were supplied by Merck. Na^+^MMT was supplied as powder by Southern Clay Products, Inc. (Texas, USA). The Department of Microbiology (SRM University, India) provided the standard cultures of the organisms. All the chemicals and reagents were used without further purification. Double-distilled water was used for the preparation of all solutions.

### Solutions Preparation

50 mL of chitosan solution (1 % wt/v in acetic acid) and 50 mL of PVA solution (1 % wt/v in water) (1:1) were mixed together in a 250 mL beaker and stirred at room temperature until a homogeneous solution was obtained. Then, different amounts of Na^+^MMT (1–5 wt%) nanoparticles were added to above mixture and stirring was continued for a further 6 h. Before casting, 1 mL of 2 % GA solution in water (a cross-linking agent) was added under stirring at room temperature. The solution was transferred immediately into Petri dishes (10 mm × 10 mm) and dried at room temperature. The formed cross-linked chitosan–PVA/Na^+^MMT films were washed with double-distilled water for neutralization and dried at room temperature [47, 48].

### Swelling Studies

Swelling studies were carried out according to the report of Vimala et al. [48]. The dried pre-weighed films were equilibrated in 250 mL phosphate buffer (pH 7.4) at 25 °C for 24 h and then water up-take of the films was measured for every 30 min using an analytical balance. The swelling ratio (*Q*) of the films was calculated using Eq. 1,

1 $$Q\;(\% ) = \frac{{W_{\text{s}} - W_{\text{d}} }}{{W_{\text{d}} }} \times 100,$$

where *W*_s_ is the weight of the swollen film at different time intervals and *W*_d_ is the weight of the dry film.

### Characterization

Bruker D8 advanced refractometer X-ray diffraction (XRD) was used to determine the intercalation or exfoliation (or both) of nanocomposite films. Transition electron microscope (TEM) images were recorded using a Tecnai F 12 JEOL-JEM 2100 at an accelerating voltage of 15 kV. The morphologies of the bio-nanocomposite films were observed by scan electron microscopy (SEM, JEOL FESEM JSM-7600F) equipped with energy-dispersive X-ray spectroscopy. Fourier transform infra-red (FTIR) spectroscopy measurement was carried on Perkin-Elmer UATR two using diamond Zn/Se plate. Small amount of sample was pressed on a Zn/Se plate and the spectra were recorded over a range of 550–4000 cm^−1^. Thermal studies of the films were carried out using TGA 7 instrument (Perkin-Elmer) at a heating rate of 10 °C min^−1^ under a constant nitrogen flow of 20 mL min^−1^.

### 5-Fluorouracil Loading and Encapsulation Efficiency

5-FU was loaded into the chitosan–PVA/Na^+^MMT nanocomposite films by the swelling method. The films (100 mg) were allowed to swell in 40 mL 5-FU solution (5 mg 5-FU, 8 mL acetone and 12 mL distilled water) for 24 h at 25 °C. The loading efficiency of 5-FU in the nanocomposite films was determined spectrophotometrically [39, 48]. The drug-loaded films were placed in 50 mL phosphate buffer solution and stirred vigorously for 4 days in order to extract the drug from the films. The solution was filtered and assayed using UV spectrophotometer at fixed *λ*_max_ value of 266 nm. The results of the drug loading and encapsulation efficiency were calculated using Eqs. 2 and 3, respectively.

2 $${\text{Drug}}\;{\text{loading}}\;(\% ) = \frac{{{\text{Weight}}\;{\text{of}}\;{\text{drug}}\;{\text{in}}\;{\text{film}} - {\text{Weight}}\;{\text{of}}\;{\text{the}}\;{\text{film}}}}{{{\text{Weight}}\;{\text{of}}\;{\text{the}}\;{\text{film}}}} \times 100,$$

3 $$ {\text{Encapsulation}}\;{\text{efficiency}}\;(\% ) = \frac{{{\text{\% }}\;{\text{actual}}\;{\text{loading}}}}{{{\text{\% }}\;{\text{theoretical}}\;{\text{loading}}}} \times 100. $$

### Release of 5-FU

For the control release studies of 5-FU from the loaded nanocomposite films, known weights were placed in a measured volume (50 mL) of 7.4 pH phosphate buffer solution at room temperature and the released amount of 5-FU was determined at different time intervals by recording the absorbance of the release medium using the UV–vis spectrophotometer [48]. The recorded absorbance was then related to the amount of 5-FU released using a calibration plot. The absorption of the solutions of 5-FU was measured at *λ*_max_ value of 266 nm.

### Antimicrobial Activity

The antibacterial activity of the nanocomposites was investigated by a disk method and the standard procedure was described elsewhere [49]. Nutrient agar medium was prepared by mixing peptone (5.0 g), beef extract (3.0 g), and sodium chloride (NaCl, 5.0 g) in 1000 mL distilled water and the pH was adjusted to 7.0. Finally, agar (15.0 g) was added to the solution. The agar medium was sterilized in an autoclave at a pressure of 6.8 kg m^−2^ (15 lbs) for 30 min. This medium was transferred into sterilized Petri dishes in a laminar air flow chamber (Microfilt Laminar Flow Ultra Clean Air Unit, Mumbai, India). After solidification of the media, bacteria (*Salmonella*, *Staphylococcus aureus*, *Streptococcus mutants* and *Escherichia coli*) (50 µL) culture were spread on the solid surface of the media. Over the inoculated Petri dish, one drop of gel solution (20 mg/10 mL distilled water) was added using a 10 µL tip and the plates were incubated for 48 h at 37 °C.

## Results and Discussion

**Fig. 2:**
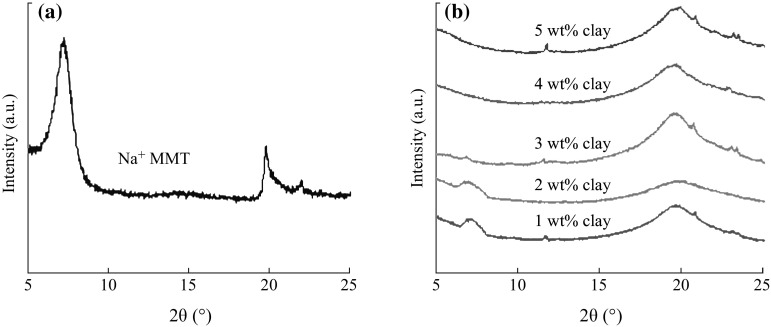
XRD patterns of Na^+^MMT and chitosan–PVA/Na^+^MMT nanocomposite films with Na^+^MMT ranging from 1 to 5 wt%

**Fig. 3:**
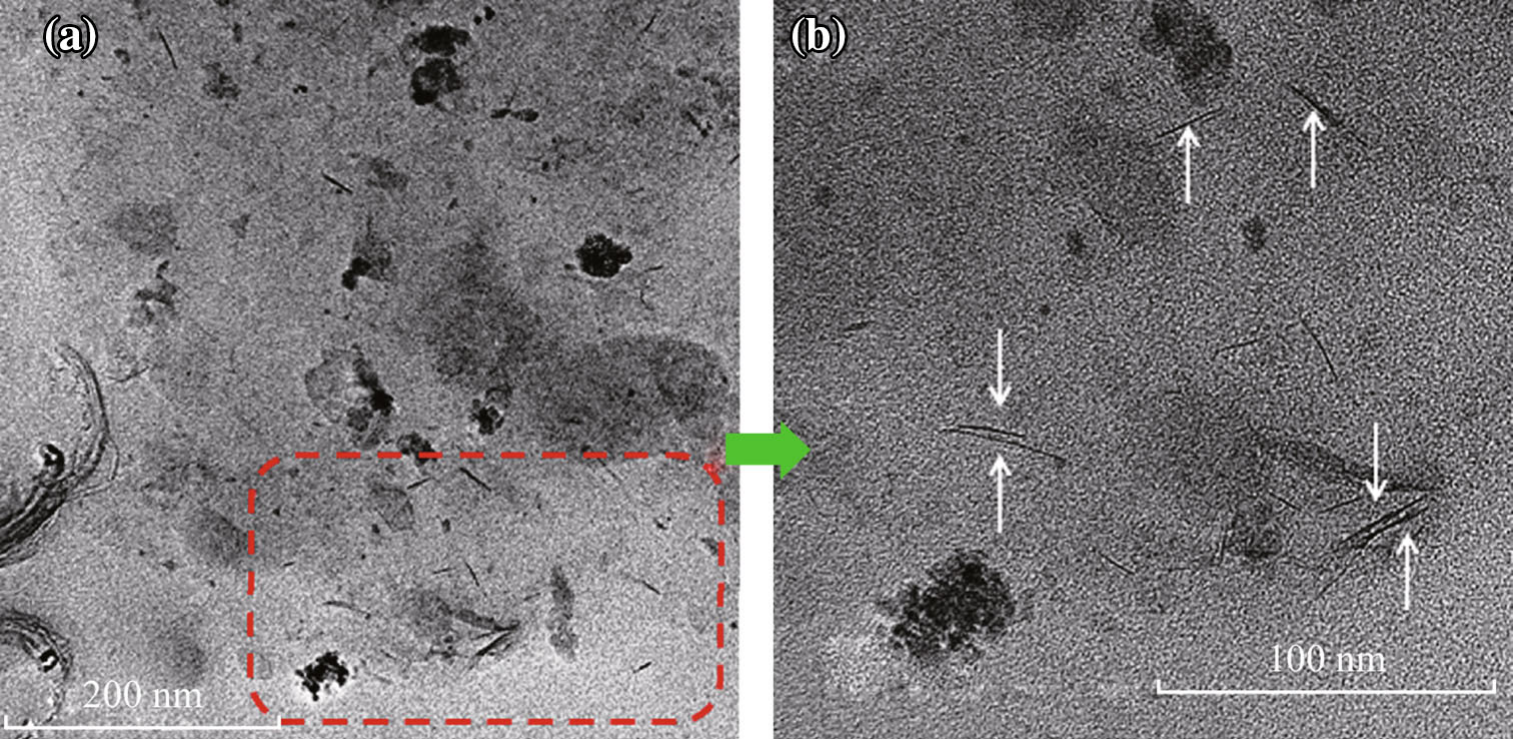
TEM images of an intercalated/exfoliated chitosan–PVA/Na^+^MMT nanocomposite (4 wt% Na^+^MMT)

The physical status of Na^+^MMT and chitosan–PVA in the synthesized chitosan–PVA/Na^+^MMT nanocomposite was studied with the help of XRD. The characteristic diffraction peak of pristine Na^+^MMT is appeared at 7.2°, corresponding to the (001) plane with a *d*-spacing of 12.1 Å (see Fig. 2a). An decrease in the intensity of the (001) plane along with a shift in the 2*θ* value from 7.2° to 7.0° was observed in the case of chitosan–PVA/Na^+^MMT nanocomposites (1 wt% clay; see Fig. 2b). The hump in the background from 18° to 24° is due to the presence of a polymer within the chitosan–PVA/Na^+^MMT nanocomposites. According to Bragg’s law, a shift in 2*θ* value from higher diffraction angle to lower diffraction angle is indicative of an increase in *d*-spacing [14, 15, 25]. The *d*-spacing data from 12.1 to 14.4 Å are given in Table 1. The increase of 2.15 Å is attributed to the co-existence of intercalation/exfoliated of chitosan–PVA within the Na^+^MMT. This is further supported by the HRTEM image (see Fig. 3) in which the presence of expanded and uniformly spaced Na^+^MMT layers in the chitosan–PVA is shown clearly. These results confirm that the Na^+^MMT resides in or entered into the chitosan–PVA matrices [50].

**Table 1:** XRD data of Na^+^MMT and chitosan/PVA–Na^+^MMT nanocomposite films

Specimen IDs	2*θ* (°)	*d*-spacing (Å)
Na^+^MMT	7.25	12.1
1 wt% clay	7.05	12.5
2 wt% clay	6.95	12.7
3 wt% clay	6.78	12.9
4 wt% clay	6.42	13.8
5 wt% clay	6.12	14.4

The TEM image of nanocomposite (5 wt% Na^+^MMT) is illustrated in Fig. 3. The images show typical morphology of layered materials, in which the dark lines correspond to Na^+^MMT clay layers, while bright areas represent the chitosan–PVA polymer matrices. As seen in Fig. 3, the interlayer distance of clay was obviously enlarged, following the addition of the mixture of chitosan–PVA polymers. Moreover, as seen from the higher magnification image (100 nm), layered silicates were exfoliated and uniformly dispersed in chitosan–PVA polymer matrices at nano-level and it is supported from the XRD patterns.

**Fig. 4:**
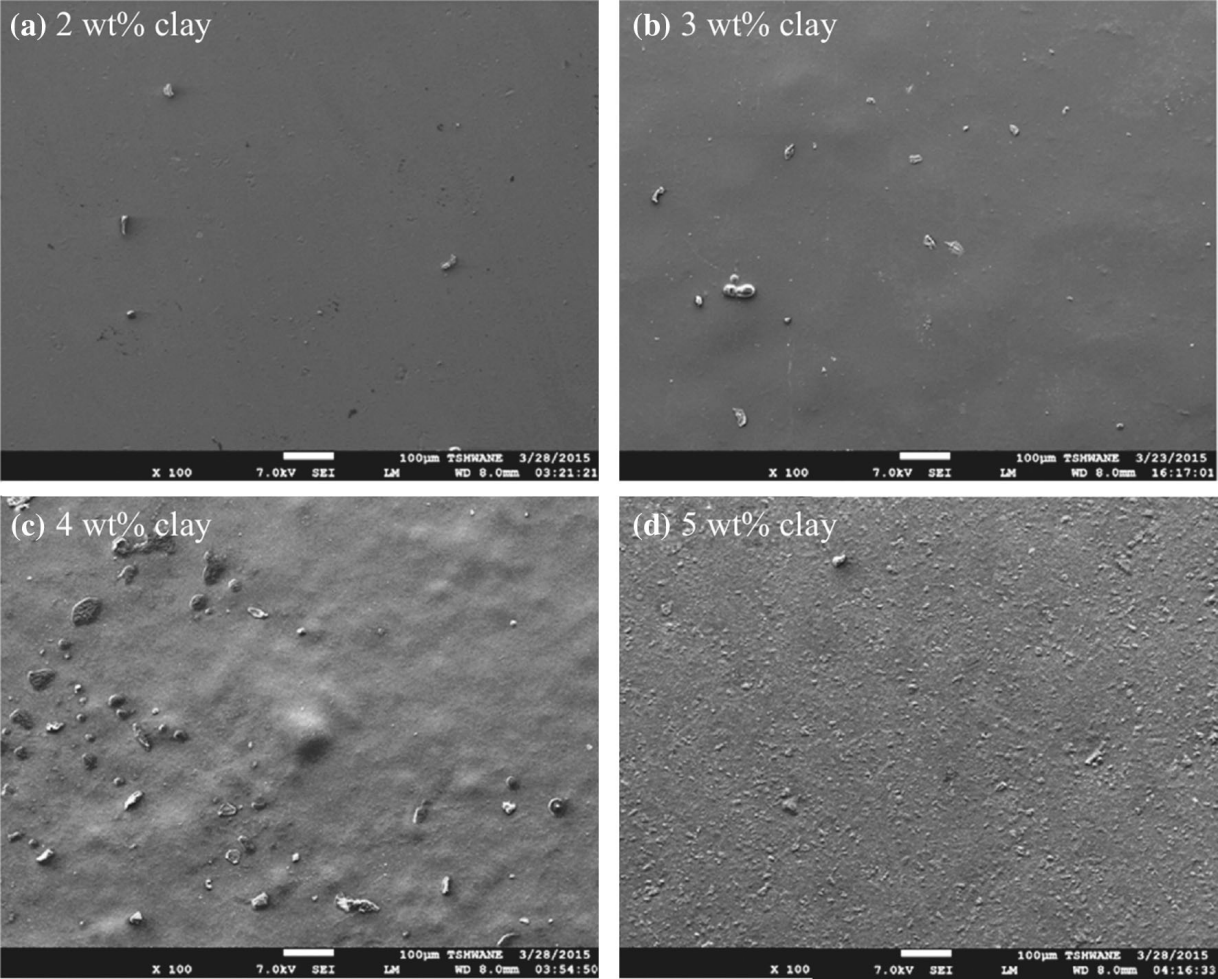
SEM images of chitosan–PVA/Na^+^MMT nanocomposite films with different Na^+^MMT contents

SEM images of the nanocomposite films are shown in Fig. 4. As seen in the figure, the surface roughness of the films was enhanced by increasing the Na^+^MMT content. Furthermore, there is a uniform surface roughness observed for 5 wt% Na^+^MMT films. The possible reason is that the polycation ability of the matrix was attracted to the negative ability of clay and resulted in a physical bond (hydrogen bond) form between them. Such exfoliated structure was confirmed in above XRD measurements and it was also observed by Rhim et al. [49].

**Fig. 5:**
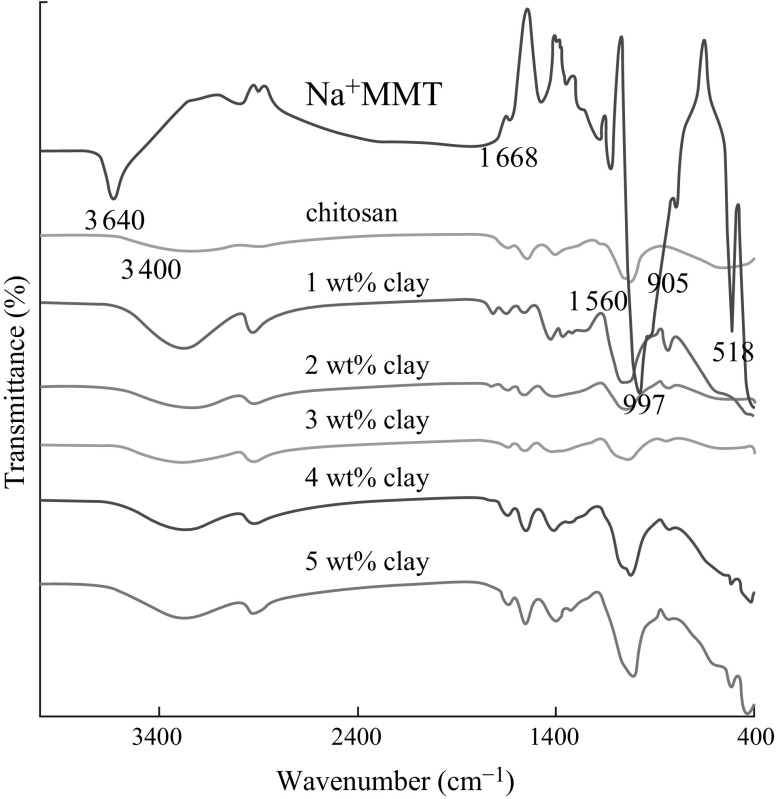
FTIR spectra of neat chitosan, Na^+^MMT, and chitosan–PVA/Na^+^MMT nanocomposite films with different Na^+^MMT contents

FTIR spectroscopy depicted in Fig. 5 was used to identify the chemical groups of polymers. From the spectrum of neat chitosan, the absorption peaks around the wave number of 3900 and 1405 cm^−1^ could be ascribed to the stretching and bending vibrations of the –NH or –OH groups. The peaks around 905 and 1560 cm^−1^ correspond to saccharide structure [51]. However, in the case of Na^+^MMT, the four types of vibrations are exhibited at 3640, 1668, 997, and 516 cm^−1^ and they are ascribed to Si–O and OH bonds, which is in good agreement with other report [50]. The spectra of the nanocomposite films include major characteristic peaks of chitosan, PVA, and Na^+^MMT. The peak around 3320 cm^−1^ is relevant to the overlapping of the –NH and –OH stretching vibrations in chitosan and PVA. Major absorptions at 425 and 524 cm^−1^ were observed in the spectra of pure Na^+^MMT and chitosan–PVA/Na^+^MMT 1–5 %, while these peaks are absent in the spectrum of neat chitosan–PVA films (not included here). These results is an evidence of the presence of Na^+^MMT nanoclays inside the chitosan–PVA matrices.

**Fig. 6:**
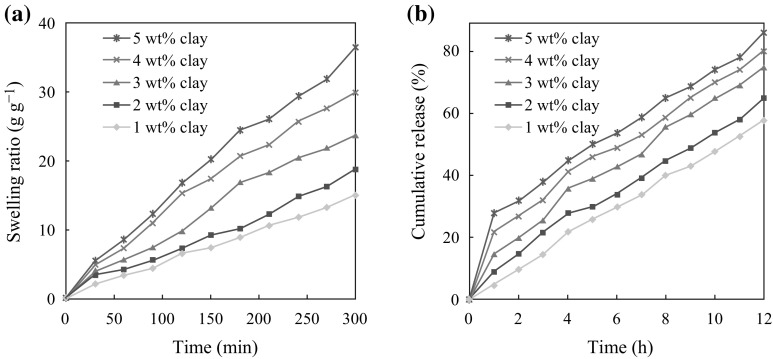
**a** Swelling capacity of chitosan–PVA/Na^+^MMT nanocomposite films with different Na^+^MMT contents. **b** 5-Fluorouracil release profile from nanocomposite films with different Na^+^MMT contents

The swelling ability of antibacterial nanocomposite films plays a significant role in their wound healing capacity and antibacterial activity in their biomedical applications due to their high water clenching capacity [48]. They can further absorb a significant amount of the wound exudates by swelling in fast curing of the wound. Figure 6a shows the swelling capacity as a function of time of the nanocomposite films developed in this study. The 5 wt% Na^+^MMT film shows the highest swelling capacity than other films. This may be due to the intermolecular interaction between water molecules in clay galleries and the lone pair electrons of –NH_2_ and –OH groups present in chitosan–PVA chains.

Drug release behavior of chitosan–PVA/Na^+^MMT films was also studied in PBS solution of pH 7.4. Figure 6b shows the % cumulative drug release behavior of the nanocomposites. It can be observed that the loading efficiency increases with increasing clay content from 1 to 5 wt% (see Table 2). This excessive increase of encapsulation may be attributed to the cationic nature of 5-FU (–NH group turn into –NH^+^ while dissolving in water) in the nanocomposites, which could enhance the interaction of 5-FU with negatively charged Na^+^MMT and chitosan–PVA polymer matrices, resulting in high encapsulation efficiency [52]. Therefore and correspondingly so, the release efficiency of 5-FU from nanocomposite films also increases with increasing the clay content.

**Table 2:** Drug loading efficiency of chitosan/PVA–Na^+^MMT nanocomposite films

Specimen IDs	Weight of the film (mg)	Weight of drug in film (mg)	% Drug loading efficiency
1 wt% clay	100	135	35
2 wt% clay	100	141	41
3 wt% clay	100	153	53
4 wt% clay	100	158	58
5 wt% clay	100	165	65

**Fig. 7:**
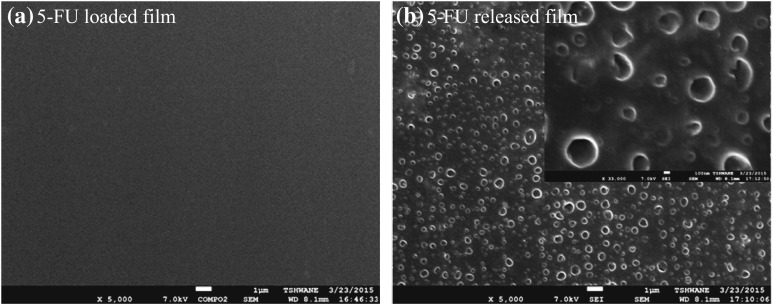
SEM images of chitosan/PVA–Na^+^MMT nanocomposites film before and after 5-FU release

However, in order to support above statements, we compare the SEM morphology of nanocomposite films before and after drug release. As shown in Fig. 7, 5-FU-loaded film shows very smooth surface, which may be due that 5-FU entered and sit on between clay galleries. In contrast, after the drug release from nanocomposite, very clear holes appear on the surface of the matrix. This is an evidence that 5-FU was released in a control manner from matrix.

**Fig. 8:**
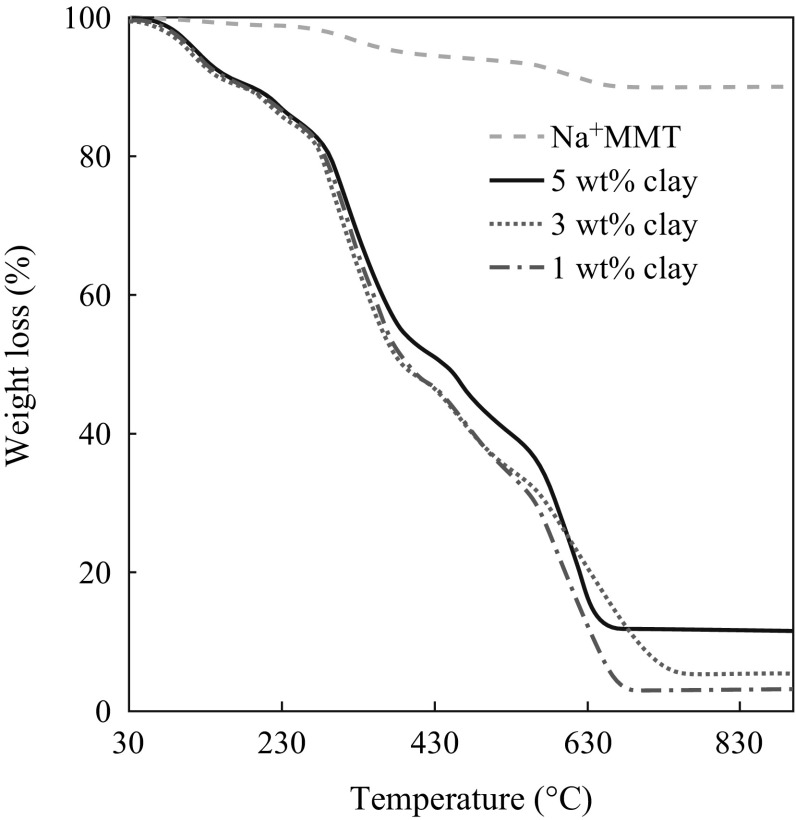
TGA curve of Na^+^MMT and chitosan/PVA–Na^+^MMT nanocomposite films with different Na^+^MMT contents

TGA curves of selected samples are shown in Fig. 8. It can be seen that Na^+^MMT shows 10 % weight loss below 100 °C due to the loss of water molecules. The nanocomposite films have higher thermal response than those of the neat chitosan, PVA or blended film. The onset temperature for decomposition shifts towards higher temperatures as the clay loading increases. This is because the presence of the clay immobilizes the polymer chains and free radicals formed during the degradation process (thus slowing down this process), or the presence of clay hinders the diffusion of volatile products through the nanocomposites (thus retarding the mass loss from the nanocomposites during degradation [53]). The increase in the thermal stability can also be attributed to the restricted thermal motions of the polymer chains inside the clay galleries [54].

**Fig. 9:**
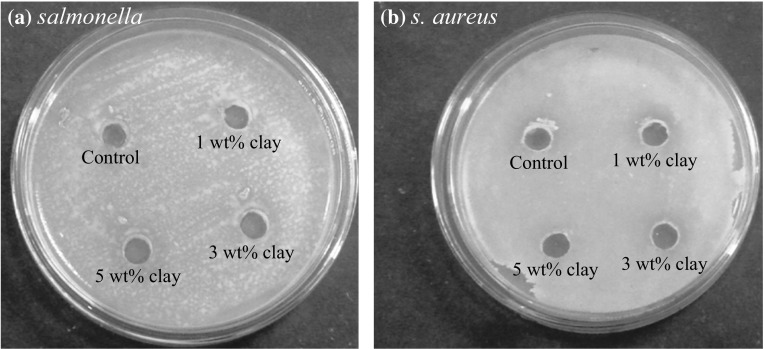
Antibacterial activity of chitosan/PVA–Na^+^MMT nanocomposite films against **a** *Salmonella* and **b** *Staphylococcus aureus*

Antimicrobial activity of Na^+^MMT-containing chitosan–PVA films were tested against *Salmonella*, *S. aureus*, *E. coli* and *S. mutants*. Figure 9 shows the characteristic test results of nanocomposite films by a disk-diffusion method. The antimicrobial activity determined by the diameter of the growth inhibition zone was dependent on the test nanocomposite film. The tests on all samples were repeated using four other microorganisms. Generally, chitosan and chitosan/Na^+^MMT nanocomposite films did not show clear microbial inhibition zones [49], whereas chitosan–PVA/Na^+^MMT nanocomposite films exhibited distinctive microbial inhibition zones against two test microorganisms (*Salmonella* and *S. aureus*) in the disk method. It is well known that chitosan itself has antimicrobial activity due to its cationic property [49]. This apparently different result for chitosan film is mainly due to the limits of detection of antimicrobial activity when using the disk method [49]. The appearance and size of the clear zone in the disk method are mainly dependent on the ratio of disk area and size of contact area, inoculum, and type of solid medium. Of interest, Na^+^MMT-incorporated nanocomposite film exhibited antimicrobial activity against the two bacteria, *Salmonella* and *S. aureus*, but did not show any antimicrobial activity against *S. mutants* and *E. coli*. Therefore, the present nanocomposite films exhibited antimicrobial activity against Gram-positive and Gram-negative bacteria. Furthermore, in the present study, the addition of Na^+^MMT to the formulations slightly condensed the antimicrobial effect of the nanocomposite films. This may be ascribed to the high cross-linking level of chitosan–PVA/Na^+^MMT nanocomposites that intercepts the diffusion of the 5-FU drug through the agar medium. On the other hand, weak inhibition of *S. aureus* growth was observed by 5 wt% clay loading nanocomposite film, having higher concentration of Na^+^MMT that is maybe due to higher rate of 5-FU drug diffusion. This is in good agreement with the data mentioned in the drug release section, indicating formulations with high clay content exhibited higher rate of drug release in the dissolution medium than those with low content of clay [50, 55].

## Conclusions

Bio-nanocomposite films-based chitosan–PVA/Na^+^MMT were developed by a simple solution casting technique. It was found that the biodegradable polymer was successfully intercalated into clay galleries. Drug loading efficiency of chitosan–PVA/Na^+^MMT films increases with increasing clay content and higher clay content exhibited higher rate of drug release. Compared with chitosan and chitosan/Na^+^MMT nanocomposite films which did not show clear microbial inhibition zones, chitosan–PVA/Na^+^MMT nanocomposite films showed distinctive microbial inhibition zones against two test microorganisms and exhibited antimicrobial activity against Gram-positive and Gram-negative bacteria. The present study is useful in developing novel antimicrobial agents for applications in wound burns/dressing, antimicrobial packaging, and prevention/treatment of infections due to the control release ability. In addition, the chitosan–PVA/Na^+^MMT nanocomposite films are a promising material for the development of new membranes for waste water treatment.

## References

[1] Uhrich KE, Cannizzaro SM, Langer RS, Shakesheff KM (1999). Polymeric systems for controlled drug release. Chem. Rev..

[2] Aguzzi C, Cerezo P, Viseras C, Caramella C (2007). Use of clays as drug delivery systems: possibilities and limitations. Appl. Clay Sci..

[3] Rinaudo M (2006). Chitin and chitosan: properties and applications. Prog. Polym. Sci..

[4] Madihally SV, Matthew HWT (1999). Porous chitosan scaffolds for tissue engineering. Biomaterials.

[5] Croisier F, Jérôme C (2013). Chitosan-based biomaterials for tissue engineering. Eur. Polym. J..

[6] Aminabhavi TM, Naik HG (1998). Chemical compatibility study of geomembranes—sorption/desorption, diffusion and swelling phenomena. J. Hazard. Mater..

[7] O’Sullivan ES, Vegas A, Anderson DG, Weir GC (2011). Islets transplanted in immunoisolation devices: a review of the progress and the challenges that remain. Endocr. Rev..

[8] Chen D-H, Leu J-C, Huang T-C (1994). Transport and hydrolysis of urea in a reactor–separator combining an anion-exchange membrane and immobilized urease. J. Chem. Technol. Biotechnol..

[9] Sabaa MW, Abdallah HM, Mohamed NA, Mohamed RR (2015). Synthesis, characterization and application of biodegradable crosslinked carboxymethyl chitosan/poly(vinyl alcohol) clay nanocomposites. Mater. Sci. Eng. C.

[10] Li JK, Wang N, Wu XS (1998). Poly(vinyl alcohol) nanoparticles prepared by freezing–thawing process for protein/peptide drug delivery. J. Control. Release.

[11] Yoshii F, Makuuchi K, Darwis D, Iriawan T, Razzak MT, Rosiak JM (1995). Heat resistance poly(vinyl alcohol) hydrogel. Radiat. Phys. Chem..

[12] Stauffer SR, Peppast NA (1992). Poly(vinyl alcohol) hydrogels prepared by freezing–thawing cyclic processing. Polymer.

[13] Bolto B, Tran T, Hoang M, Xie Z (2009). Crosslinked poly(vinyl alcohol) membranes. Prog. Polym. Sci..

[14] Joshi GV, Kevadiya BD, Patel HA, Bajaj HC, Jasra RV (2009). Montmorillonite as a drug delivery system: intercalation and in vitro release of timolol maleate. Int. J. Pharm..

[15] Joshi GV, Patel HA, Kevadiya BD, Bajaj HC (2009). Montmorillonite intercalated with vitamin B1 as drug carrier. Appl. Clay Sci..

[16] Bergaya F, Lagaly G (2013). Handbook of Clay Science.

[17] Chen Y, Zhou A, Liu B, Liang J (2010). Tramadol hydrochloride/montmorillonite composite: preparation and controlled drug release. Appl. Clay Sci..

[18] Iliescu RI, Andronescu E, Voicu G, Ficai A, Covaliu CI (2011). Hybrid materials based on montmorillonite and cytostatic drugs: preparation and characterization. Appl. Clay Sci..

[19] Feng S-S, Mei L, Anitha P, Gan CW, Zhou W (2009). Poly(lactide)–vitamin E derivative/montmorillonite nanoparticle formulations for the oral delivery of Docetaxel. Biomaterials.

[20] Dong Y, Feng S-S (2005). Poly(d,l-lactide-*co*-glycolide)/montmorillonite nanoparticles for oral delivery of anticancer drugs. Biomaterials.

[21] Lee Y-H, Kuo T-F, Chen B-Y, Feng Y-K, Wen Y-R, Lin W-C, Lin FH (2005). Toxicity assessment of montmorillonite as a drug carrier for pharmaceutical applications: yeast and rats model. Biomed. Eng..

[22] Lin FH, Lee YH, Jian CH, Wong J-M, Shieh M-J, Wang C-Y (2002). A study of purified montmorillonite intercalated with 5-fluorouracil as drug carrier. Biomaterials.

[23] Nunes CD, Vaz PD, Fernandes AC, Ferreira P, Romão CC, Calhorda MJ (2007). Loading and delivery of sertraline using inorganic micro and mesoporous materials. Eur. J. Pharm. Biopharm..

[24] Seki Y, Yurdakoç K (2006). Adsorption of promethazine hydrochloride with KSF montmorillonite. Adsorption.

[25] Joshi GV, Kevadiya BD, Bajaj HC (2010). Design and evaluation of controlled drug delivery system of buspirone using inorganic layered clay mineral. Microporous Mesoporous Mater..

[26] E. Healey, G.e. Stillfried, S. Eckermann, J.p. Dawber, P.r. Clingan, M. Ranson, Comparative effectiveness of 5-fluorouracil with and without oxaliplatin in the treatment of colorectal cancer in clinical practice. Anticancer Res. **33**(3), 1053–1060 (2013), http://ar.iiarjournals.org/content/33/3/1053.long 23482781

[27] Osaki M, Tatebe S, Goto A, Hayashi H, Oshimura M, Ito H (1997). 5-Fluorouracil (5-FU) induced apoptosis in gastric cancer cell lines: role of the p53 gene. Apoptosis.

[28] Cameron DA, Gabra H, Leonard RC (1994). Continuous 5-fluorouracil in the treatment of breast cancer. Br. J. Cancer.

[29] Chen H, Wu W, Li Y, Gong T, Sun X, Zhang Z (2014). A novel brain targeted 5-FU derivative with potential antitumor efficiency and decreased acute toxicity: synthesis, in vitro and in vivo evaluation. Die Pharmazie.

[30] Ostertag D, Amundson KK, Lopez Espinoza F, Martin B, Buckley T (2012). Brain tumor eradication and prolonged survival from intratumoral conversion of 5-fluorocytosine to 5-fluorouracil using a nonlytic retroviral replicating vector. Neurooncology.

[31] van Riel JMGH, van Groeningen CJ, Albers SHM, Cazemier M, Meijer S, Bleichrodt R, van den Berg FG, Pinedo HM, Giaccone G (2000). Hepatic arterial 5-fluorouracil in patients with liver metastases of colorectal cancer: single-centre experience in 145 patients. Ann. Oncol..

[32] Cascinu S, Silva RR, Barni S, Labianca R, Frontini L (1999). A combination of gemcitabine and 5-fluorouracil in advanced pancreatic cancer, a report from the Italian Group for the Study of Digestive Tract Cancer (GISCAD). Br. J. Cancer.

[33] Rao S, Cunningham D (2002). Advanced pancreatic cancer—5 years on. Ann. Oncol..

[34] W.H. Isacoff, H.A. Reber, F.M. Purcell, B.M. Clerkin, K.M. Clerkin, Low-dose continuous infusion 5-fluorouracil combined with weekly leucovorin, nab-paclitaxel, oxaliplatin, and bevacizumab for patients with advanced pancreatic cancer: a pilot study. J. Clin. Oncol. (Meet. Abstr.) **28**(15), e14545 (2010), http://meeting.ascopubs.org/cgi/content/abstract/28/15_suppl/e14545

[35] Nakano J, Huang C, Liu D, Masuya D, Nakashima T, Yokomise H, Ueno M, Wada H, Fukushima M (2006). Evaluations of biomarkers associated with 5-FU sensitivity for non-small-cell lung cancer patients postoperatively treated with UFT. Br. J. Cancer.

[36] Zhao J-G, Ren K-M, Tang J (2014). Overcoming 5-FU resistance in human non-small cell lung cancer cells by the combination of 5-FU and cisplatin through the inhibition of glucose metabolism. Tumor Biol..

[37] Lynch TJ, Kass F, Elias AD, Skarin A, Iii EF, Kalish LA, Strauss G, Shulman LN, Sugarbaker DJ (1993). Cisplatin, 5-fluorouracil, and etoposide for advanced non-small cell lung cancer. Cancer.

[38] Pan X, Zhang X, Sun H, Zhang J, Yan M, Zhang H (2013). Autophagy inhibition promotes 5-fluorouracil-induced apoptosis by stimulating ROS formation in human non-small cell lung cancer A549 cells. PLoS One.

[39] Kevadiya BD, Patel TA, Jhala DD, Thumbar RP, Brahmbhatt H (2012). Layered inorganic nanocomposites: a promising carrier for 5-fluorouracil (5-FU). Eur. J. Pharm. Biopharm..

[40] B. Van Triest, H.M. Pinedo, G. Giaccone, G.J. Peters, Downstream molecular determinants of response to 5-fluorouracil and antifolate thymidylate synthase inhibitors. Ann. Oncol. **11**(4), 385–391 (2000), http://annonc.oxfordjournals.org/content/11/4/385 10.1023/a:1008351221345 10847455

[41] Yohan D, Cruje C, Lu X, Chithrani D (2014). Elucidating the uptake and distribution of nanoparticles in solid tumors via a multilayered cell culture model. Nano–Micro Lett..

[42] Aranda E, Díaz-Rubio E, Cervantes A, Antón-Torres A, Carrato A (1998). Randomized trial comparing monthly low-dose leucovorin and fluorouracil bolus with weekly high-dose 48-hour continuous-infusion fluorouracil for advanced colorectal cancer: a Spanish Cooperative Group for Gastrointestinal Tumor Therapy (TTD) study. Ann. Oncol..

[43] R.B. Diasio, Z. Lu, Dihydropyrimidine dehydrogenase activity and fluorouracil chemotherapy. J. Clin. Oncol. **12**(11), 2239–2242 (1994), http://jco.ascopubs.org/content/12/11/2239 10.1200/JCO.1994.12.11.2239 7964937

[44] Gamelin EC, Danquechin-Dorval EM, Dumesnil YF, Maillart PJ, Goudier MJ (1996). Relationship between 5-fluorouracil (5-FU) dose intensity and therapeutic response in patients with advanced colorectal cancer receiving infusional therapy containing 5-FU. Cancer.

[45] Meropol NJ, Niedzwiecki D, Hollis D, Schilsky RL, Mayer RJ, Cancer and The Leukemia Group B (2001). Phase II study of oral eniluracil, 5-fluorouracil, and leucovorin in patients with advanced colorectal carcinoma. Cancer.

[46] Li S, Wang A, Jiang W, Guan Z (2008). Pharmacokinetic characteristics and anticancer effects of 5-fluorouracil loaded nanoparticles. BMC Cancer.

[47] Parida ANU, Binhani B, Nayak P (2011). Synthesis and characterization of chitosan–polyvinyl alcohol blended with cloisite 30B for controlled release of the anticancer drug curcumin. J. Biomater. Nanobiotechnol..

[48] Vimala MYK, Varaprasad K, Reddy N, Ravindra S, Naidu N, Raju K (2011). Fabrication of curcumin encapsulated chitosan–PVA silver nanocomposite films for improved antimicrobial activity. J. Biomater. Nanobiotechnol..

[49] Rhim J-W, Hong S-I, Park H-M, Ng PKW (2006). Preparation and characterization of chitosan-based nanocomposite films with antimicrobial activity. J. Agric. Food Chem..

[50] Koosha M, Mirzadeh H, Shokrgozar MA, Farokhi M (2015). Nanoclay-reinforced electrospun chitosan/PVA nanocomposite nanofibers for biomedical applications. RSC Adv..

[51] Shen G, Guo Y, Sun X, Wang X (2014). Electrochemical aptasensor based on prussian blue-chitosan–glutaraldehyde for the sensitive determination of tetracycline. Nano–-Micro Lett..

[52] Seema MD (2013). Clay–polymer nanocomposites as a novel drug carrier: synthesis, characterization and controlled release study of propranolol hydrochloride. Appl. Clay Sci..

[53] Costache MC, Wang D, Heidecker MJ, Manias E, Wilkie CA (2006). The thermal degradation of poly(methyl methacrylate) nanocomposites with montmorillonite, layered double hydroxides and carbon nanotubes. Polym. Adv. Technol..

[54] Kaci M, Remili C, Benhamida A, Bruzaud S, Grohens Y (2012). Recyclability of polystyrene/clay nanocomposites. Mol. Cryst. Liq. Cryst..

[55] M. Kouchak, A. Ameri, B. Naseri, S. Kargar Boldaji, Chitosan and polyvinyl alcohol composite films containing nitrofurazone: preparation and evaluation. Iran. J. Basic Med. Sci. **17**(1), 14–20 (2014), http://www.ncbi.nlm.nih.gov/pmc/articles/PMC3938881/pdf/ijbms-17-014 PMC3938881 24592302

